# SERPINB1 as a critical regulator of inflammation and protease activity in human monocytic cells

**DOI:** 10.1093/immhor/vlag053

**Published:** 2026-09-24

**Authors:** Jareb J Pérez-Caraballo, Xiang Ye, Jian Cui, Rubén Martínez-Barricarte

**Affiliations:** Division of Genetic Medicine and Clinical Pharmacology, Department of Medicine, Vanderbilt University Medical Center, Nashville, TN, United States; Division of Molecular Pathogenesis, Department of Pathology, Microbiology, and Immunology, Vanderbilt University Medical Center, Nashville, TN, United States; Division of Genetic Medicine and Clinical Pharmacology, Department of Medicine, Vanderbilt University Medical Center, Nashville, TN, United States; Division of Molecular Pathogenesis, Department of Pathology, Microbiology, and Immunology, Vanderbilt University Medical Center, Nashville, TN, United States; Division of Genetic Medicine and Clinical Pharmacology, Department of Medicine, Vanderbilt University Medical Center, Nashville, TN, United States; Division of Molecular Pathogenesis, Department of Pathology, Microbiology, and Immunology, Vanderbilt University Medical Center, Nashville, TN, United States; Division of Genetic Medicine and Clinical Pharmacology, Department of Medicine, Vanderbilt University Medical Center, Nashville, TN, United States; Division of Molecular Pathogenesis, Department of Pathology, Microbiology, and Immunology, Vanderbilt University Medical Center, Nashville, TN, United States; Vanderbilt Center for Immunobiology, Vanderbilt University Medical Center, Nashville, TN, United States; Vanderbilt Genetics Institute, Vanderbilt University Medical Center, Nashville, TN, United States; Vanderbilt Institute for Infection, Immunology and Inflammation, Vanderbilt University Medical Center, Nashville, TN, United States

**Keywords:** monocytes, proteomics, SERPINB1, serine protease inhibitors, THP-1

## Abstract

SERPINB1, a member of the serpin superfamily, functions primarily as a serine protease suicide inhibitor via its reactive center loop (RCL). While murine studies have elucidated its role in neutrophil homeostasis and survival, the functions and pathways orchestrated by human SERPINB1, particularly in monocytes, remain largely undefined. Here, we demonstrate that SERPINB1 is under purifying selection, indicative of an essential, non-redundant role in human immunity comparable to that of genes known to cause immune diseases when mutated. Flow cytometric and Western blot analyses reveal ubiquitous expression in immune cells, with monocytes exhibiting the highest levels and the most active utilization of SERPINB1. Using CRISPR/Cas9-mediated knockout in THP-1 monocytic cells, we found that SERPINB1 deficiency does not impair cell viability but significantly enhances proinflammatory responses, notably increasing IL-1β production in response to lipopolysaccharide stimulation. Bulk RNA sequencing corroborated upregulation of inflammatory genes, while activity-based proteomic profiling uncovered broad alterations in enzymatic activity, affecting pathways beyond protease regulation, including ribosomal function and cellular metabolism. These findings suggest that human SERPINB1 extends its function beyond protease inhibition to modulate monocyte inflammatory activation and metabolic processes. Further mechanistic investigations are warranted to delineate the molecular interactions underpinning SERPINB1’s regulatory network in monocytes. Yet, our study provides novel insights into SERPINB1’s multifaceted functions in human immunity and highlights its potential as a therapeutic target for controlling dysregulated inflammation.

## Introduction

**Figure 1:**
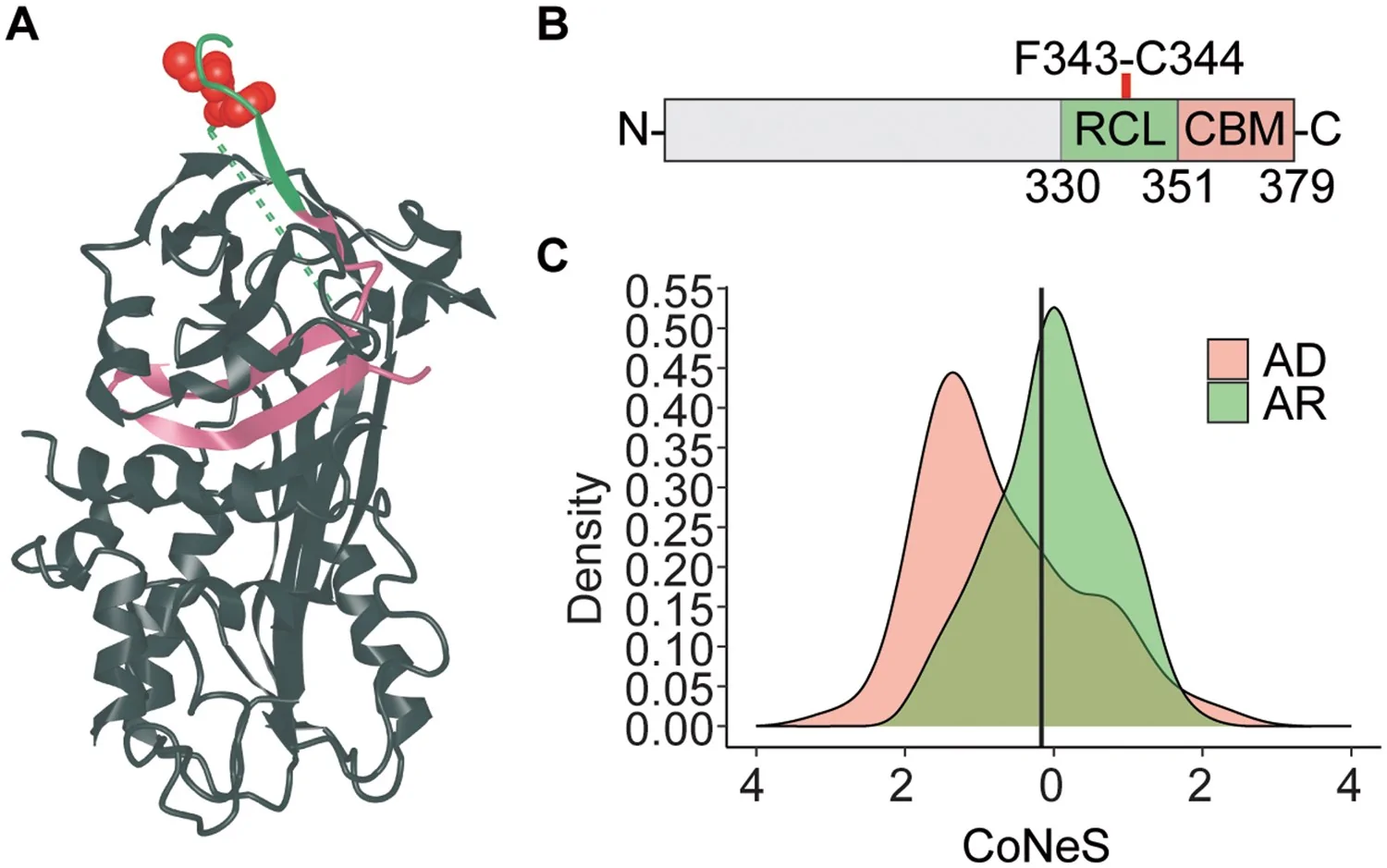
SERPINB1 structure and purifying selection. (A) Structure of SERPINB1 highlighting the active site (Red and residues shown as spheres), RCL (green) and CBM (pink). (B) Schematic representation of SERPINB1’s domains with the active site represented with a red line and the amino acids involved indicated above. RCL: Reactive center loop, CBM: CARD-Binding Motif. (C) CoNeS score of *SERPINB1* shown with a vertical line. The scores for genes that, when mutated, cause autosomal recessive (AR) and autosomal dominant (AD) inborn errors of immunity are shown.

SERPINB1 (also known as Leukocyte elastase inhibitor) is a serine protease suicide inhibitor belonging to the serpin superfamily of proteins.^1–4^ It is classified in clade B according to its phylogenetic origin, and it lacks a signal peptide, making it intracellular.^1^^,^^5^ Like most other proteins in the serpin superfamily, its secondary structure consists of 9 α-helixes, 3 β-sheets, and a flexible C-terminal region designated the reactive center loop (RCL) (Fig. 1A, B).^6^ This region confers its enzymatic inhibitory function.^6^ SERPINB1 is referred to as a suicide inhibitor because it covalently binds to its target protease. SERPINB1’s RCL contains 2 reactive sites, one at cysteine 344 and another at phenylalanine 343, and both are responsible for inhibiting different target proteases. Inhibition of the target protease is a multi-step mechanism involving cleavage of the RCL, leading to a covalent complex between the protease and SERPINB1 in which the active site of the protease is permanently inhibited.^6^ Although the main function of SERPINB1 is protease inhibition via the RCL, it also has a CARD-binding Motif (CBM) that has been shown to interact with and inhibit, in a non-covalent manner, various other proteases (Fig. 1A, B).^7^ Despite these findings, the pathways orchestrated by SERPINB1 remain poorly understood in humans.

Most of the work to unravel the function of SERPINB1 has been performed in mice. Mice have 4 isoforms of *serpinb1a* through *d.*^8^  *Serpinb1b* is primarily expressed in the brain. *Serpinb1c* contains an alternative spliced exon due to the insertion of an endogenous retrovirus-like sequence that renders it non-functional. *Serpinb1d* lacks several exons, classifying it as a pseudogene. Based on 79% sequence homology and similar expression patterns, *Serpinb1a* has been proposed as the ortholog of human *SERPINB1.*^8^ Serpinb1a is critical for neutrophil homeostasis.^9^^,^^10^ Its expression is known to increase during development, when neutrophil serine proteases are synthesized and stored in primary azurophil granules. By controlling excess intracellular protease activity, serpinb1a is critical for neutrophil survival at this stage.^10^ Furthermore, as neutrophils age, granule membrane permeabilization occurs. This results in the leakage of both cathepsin G and proteinase 3 into the cytoplasm.^11^ Serpinb1a has been shown to inhibit these proteases when they leak into the cytoplasm, thereby protecting against uncontrolled protease activity and cell death. Consistently, neutrophils deficient in Serpinb1a can undergo cell death mediated by both cathepsin G and proteinase 3.^11^^,^^12^ Cathepsin G-mediated cell death is caspase-independent, whereas proteinase 3-dependent cell death is caspase-dependent, as proteinase 3 generates caspase 3.^11^^,^^12^ Furthermore, through the CBM, SERPINB1 can inhibit caspases, suggesting that it has a dual function in preventing cell death through uncontrolled protease activity.^7^ Yet the role of SERPINB1 and the pathways it orchestrates remain elusive in most other immune cells, especially in humans. In this article, we describe, using an unbiased approach, additional functions of SERPINB1 in human monocytic cells.

## Material and methods

### Purifying selection analysis

To assess the strength of negative selection, we used the Distribution of Consensus Negative Selection (CoNeS) from Rapaport et al. for *SERPINB1* and genes known to cause inborn errors of immunity (IEI) when mutated.^13^ We also studied the f parameter for *SERPINB1* and all other genes in the genome.^13^^,^^14^

### Structural analysis of SERPINB1

The structure of SERPINB1 was downloaded from the NCBI website (PDB ID: 4GA7).^15^ Analysis and figure generation were performed using the iCn3D software.

### SERPINB1 levels by FACS

Peripheral blood mononuclear cells (PBMCs) from healthy controls were first stained with either Live/Dead Fixable Aqua (Invitrogen) or Zombie UV viability dye (BioLegend) for 20 min on ice. Cells were then washed 2 times with phosphate-buffered saline (PBS) and stained with combinations of the following antibodies as shown in Fig. S1; anti-CD3 V450 (UCHT1, BD biosciences, 1/50), anti-CD161 PerCP/Cy5.5 (HP-3G10, BioLegend, 1/50), anti-Vα7.2-TCR APC (3C10, BioLegend, 1/100), anti-Vα24Jα18-TCR PerCP/eFluor710 (6B11, eBoisciences, 1/50), anti-lineage Pacific Blue (anti-CD3/14/16/19/20/56, BioLegend, 1/100), anti-CD127 eF660 (eBioRDRS, eBiosciences, 1/50), anti-CRTH2 APC/Cy7 (BM16, BioLegend, 1/50), anti-CD45 PE (HI30, BioLegend, 1/200), anti-CD117 PE/Cy7 (104D2, eBiosciences, 1/100), anti-CCR6 BV650 (G034E3, BioLegend, 1/50), anti-CCR7 Alexa647 (G043H7, BioLegend, 1/50), anti-CD45RA APC/H7 (HI100, BD Biosciences, 1/50), anti-CXCR3 PE (G025H7, BioLegend, 1/50), anti-CD4 BUV395 (SK3, BD Biosciences, 1/50), γδ-TCR PE (B1, BioLegend, 1/50), anti-CD8 PE/Cy7 (SK1, BioLegend, 1/200), HLA-DR Pacific Blue (L243, BioLegend, 1/50), anti-CD14 BV650 (M5E2, BioLegend, 1/100), anti-CD56 APC (NCAM16.2, BD Biosciences, 1/50), anti-CD19 APC/Cy7 (HIB19, BioLegend, 1/50), anti-CD3 PE (UCHT1, BD Biosciences, 1/100), anti-CD15 PE (VIMC6, Miltenyi Biotec, 1/100), anti-CD16 PE/Cy7 (3G8, BioLegend, 1/200), anti-CD1c BV605 (L161, BioLegend, 1/50), anti-CD303 APC (AC144, Miltenyi Biotec, 1/50), anti-CD11c Alexa700 (B-ly6, BD Biosciences, 1/100), anti-CD141 PE (M80, BioLegend, 1/50) and anti-HLA-DR PE/Cy7 (G46-6, BD Biosciences, 1/100) diluted in PBS for 30 mins on ice. PBMCs were then washed twice with PBS and fixed with 1% Paraformaldehyde (Electron Microscopy Sciences) for 10 min on ice. Two washes were performed with BD Perm/Wash buffer (BD Biosciences), followed by a 45-min incubation on ice with an in-house-produced and previously validated anti-SERPINB1 antibody conjugated with FITC, kindly provided by Drs. Lifei Hou and Eileen Remold-O’Donnell (Boston Children’s Hospital) diluted 1/50.^16^ PBMCs were then washed twice with PBS, and data were acquired on an LSRII flow cytometer (BD Biosciences) using BD FACSDIVA software (BD Biosciences). Data were later analyzed using FlowJo software version 10.5.3. Flow sorting of B, NK, total monocytes, CD4^+^ T, and CD8^+^ T cells was performed following the gating strategy in Fig. S1 using a FACS Aria (BD Biosciences).

### Cell culture

THP-1 and HEK293T cells were purchased from ATCC (Manassas, Virginia, USA). THP-1 cells were cultured in RPMI 1640 (Sigma-Aldrich, Burlington, Massachusetts, USA), while HEK 293 T cells were cultured in DMEM (Sigma-Aldrich), both supplemented with 10% FBS (Avantor, Radnor, PA). All cells were maintained at 37 °C and 5% CO2.

### CRISPR/Cas9 editing

Single Guide RNAs (sgRNAs) were designed using the Broad Institute online tool (https://portals.broadinstitute.org/gppx/crispick/public/). AATGCTGATATCAACAAACGTGG was used to target exon 3, and TCTGAATGGTGCATTCGTCGTGG was used to target exon 5. GTATTACTGATATTGGTGGG was used as a non-target control. The sgRNA sequences were introduced in a lentiCRISPRv2 vector (Addgene, Watertown, Massachusetts, USA) on the Esp31 restriction site (Thermo Fisher, Waltham, Massachusetts, USA). To generate lentiviral supernatant, HEK293T cells were plated at a density of 1 × 10^5^ cells/mL, 2 mL per well, in 6-well plates. After cells reached a 70% confluency, they were transfected with 500 ng of psPAX2 plasmid (Addgene), 50 ng of pMD2.G plasmid (Addgene), and 500 ng of lentiCRISPR V2 plasmid containing the sgRNA sequences using Lipofectamine 3,000 (Thermo Fisher). After 24 h of incubation, the media was replaced. After a 2-d rest period, the cell suspension was homogenized by pipetting up and down, and the media was collected and centrifuged at 2,000× *g* for 10 min. For viral transduction, 5 × 10^5^ THP-1 cells were added to a 15 mL tube with 5 mL of RPMI 10% FBS and 8 μg/mL polybrene (Millipore-Sigma Burlington, MA) and 1% PEG (Fisher Scientific Pittsburgh, Pennsylvania, USA) and 1 mL of lentiviral supernatant. The mixture was then centrifuged at 1,000× *g* for 1 h at room temperature. The cells were then resuspended in 2 mL of media and transferred to a 6-well plate. After a 48-h rest period, 5 µg/mL of puromycin (Thermo Fisher) was added to the media. Once a polyclonal culture was established, single-cell clones were generated by plating THP-1 cells at a density of 0.5 cells/well in a flat-bottom 96-well plate. SERPINB1 knockout was validated by Sanger sequencing and Western blot.

### Western blots

Total protein lysates were prepared using RIPA buffer and quantified using the DC Assay (Bio-Rad). Briefly, 30 µg of protein was run by SDS-PAGE and transferred to a Polyvinylidene Difluoride (PDFV) membrane (Millipore-Sigma) using a Trans-Blot Turbo transfer system (Biorad). The membrane was probed with anti-SERPINB1 (generously provided by Eileen Remold-O’Donnell, Boston Children’s Hospital)^16^ and anti-GAPDH antibodies (Invitrogen, Waltham, Massachusetts, USA) as a loading control. Membranes were imaged by chemiluminescence with Pierce ECL Substrate (Thermo Fisher) in an iBright 1500 imager (Invitrogen).

### Cell death and viability assays

Cell death was evaluated by measuring lactate dehydrogenase (LDH) release using the CyQUANT LDH Cytotoxicity Assay Kit (Thermo Fisher). Cell viability was evaluated by plating 1 × 10^6^ THP-1 cells in a 6-well plate. Cells were maintained in culture for 7 d, and viability was measured daily using trypan blue (Corning, Corning, New York, USA) with the Countess 3 FL cell counter (Invitrogen). Each condition was performed in triplicate, and the percentage of live cells was averaged.

### IL-1β Secretion

Human THP-1 cells (1 × 10^6^ cells/well in a 6-well plate, in triplicate) were cultured in the presence of 100 ng of LPS for 12 h, after which the culture supernatant was collected. IL-1β concentration in the supernatant was measured using the human IL-1β ELISA kit (Invitrogen).

### Activity-based proteomics assay

THP-1 cells were lysed using Pierce IP Lysis Buffer lysis (Thermo Scientific) and quantified by DC Assay (Biorad). After that, 100 µg of total protein lysate was incubated with ActivX Desthiobiotin-FP Serine Hydrolase Probe (Thermo Scientific) following the manufacturer’s protocol. Peptides were resuspended in 0.2% FA to a concentration of 200 ng/μL. Peptide concentrations were estimated using a Nanodrop 2000 Spectrophotometer (Thermo Scientific), and samples were adjusted to 50 ng/μL. In brief, 0.015% n-dodecyl-β-d-maltoside was added to diaPASEF samples. DiaPASEF data were acquired on 150 ng of peptides using a 30-min aqueous-to-organic gradient delivered via a nanoELUTE2 on a PepSep column with a 75-μm internal diameter, 25-cm length, and 1.5-μm particle size, coupled to a timsTOF HT instrument (Bruker) with a 20-μm CaptiveSpray emitter. diaPASEF data were collected in 12 PASEF ramps from 0.75 to 1.3 1/*k*  _0_ covering 350 to 1,250 *m*/*z* via variable windows ranging in size from 12.27 to 122.81 Th with 50 ms accumulation time. Orbitrap DIA data were acquired on 150 ng of peptides using a 95 min aqueous-to-organic gradient method delivered via a Dionex Ultimate 3000 UHPLC on a 360 μm outer diameter × 100 μm internal diameter column packed with 20 cm of 3 μm C18 reverse phase material (Jupiter, Phenomenex) coupled to an Orbitrap Exploris 480 instrument (Thermo Scientific). Precursor spectra from 385 to 1,025 *m*/*z* were collected after thirty 20 *m*/*z* windows of DIA MS/MS spectra from 400 to 1,000 *m*/*z* or thirty-one 20 *m*/*z* windows of DIA MS/MS spectra from 390 to 1,010 *m*/*z*. Differentially enriched target proteins were exported from Spectronaut (version 19.9.250512) for the scatter plot and domain enrichment analysis. Domain analysis was performed with InterProScan (version 5.65–97.0). Fisher exact test was used to assess the enrichment of domains or pathways against all detected proteins as the background, with *P*-values adjusted using the Benjamini-Hochberg method. All the plots were created using the R package ggplot2. Raw data can be found in the MassIVE repository, accession number MSV000102433.

### RNAseq

Total RNA was extracted using the RNeasy Plus Mini Kit (Qiagen, Hilden, Germany) following the manufacturer’s protocol. RNA was quantified using a NanoDrop One/OneC Microvolume UV-Vis Spectrophotometer (Thermo Scientific). Samples were sequenced in multiplex paired-end 150 bp on a NovaSeq 6000 Sequencing System (Illumina, San Diego, California, USA). Transcript abundance was quantified using kallisto (v0.51.1) with pseudo-alignment to the human reference transcriptome (Homo_sapiens.GRCh38.115). Gene-level count estimates were generated using tximport (v1.36.1) in R (v4.5.3). Differential gene expression analysis was performed using DESeq2 (v1.48.2). The analysis focused on transcriptional responses to LPS stimulation and genotype-dependent effects. Specifically, comparisons were performed between LPS and Nostim conditions within each genotype, as well as between WT and KO under LPS stimulation. Genes with an adjusted *P*-value < 0.05 and absolute log2 fold change ≥ 1 were considered significantly differentially expressed. For visualization, variance-stabilized expression values were calculated using DESeq2. Heatmaps of the top 50 differentially expressed genes were generated using pheatmap (v1.0.13), with gene identifiers converted to gene symbols for clarity. Functional enrichment analysis was conducted on significantly differentially expressed genes using clusterProfiler (v4.16.0). Gene Ontology (GO) biological process enrichment and Kyoto Encyclopedia of Genes and Genomes (KEGG) pathway analysis were performed to identify pathways associated with LPS stimulation and genotype-specific responses. Enriched pathways were visualized using dot plots, and gene annotation was performed using org. Hs.eg.db (v3.22.0). The gene count matrix can be found in Table S1.

### Ethical statement

The study received approval from the Vanderbilt University Medical Center Human Research Protection Program. Informed consent was obtained from all participants, and all procedures were conducted in accordance with the ethical principles set forth in the Declaration of Helsinki.

## Results

### *SERPINB1* is under purifying selection

We first hypothesized that *SERPINB1* plays a critical role in the human immune system. To test this hypothesis, we evaluated evolutionary constraints on *SERPINB1* by examining its purifying (negative) selection metrics. Purifying selection is the evolutionary process by which deleterious alleles in critical genes are removed from populations because they confer a survival disadvantage. Hence, genes under strong purifying selection are intolerant of genetic variation, indicating a critical, non-redundant function in human physiology and survival. To assess purifying selection in *SERPINB1,* we used CoNeS, which integrates several methods to quantify purifying selection and is the most reliable for deciphering evolutionary constraints in immune genes.^13^ Our analysis shows that *SERPINB1* has a CoNeS score of −0.17, consistent with purifying selection. To put this score into perspective, we compared *SERPINB1’s* CoNeS score with all genes known to cause inborn errors of immunity (IEIs) when mutated. IEIs are a group of monogenic diseases that alter the development and/or function of the immune system, causing susceptibility to severe infection, autoimmunity, or cancer, among others.^17^^,^^18^ IEI-causing genes are known to be under strong purifying selection because they cause diseases of high mortality and morbidity.^13^^,^^19^^,^^20^ When we compared *SERPINB1’s* CoNeS score with all IEI-causing genes, we found that it falls within the set of genes that cause an autosomal recessive disease when mutated (Fig. 1C). To validate the results obtained with CoNeS, we assessed the f-parameter for *SERPINB1* as an additional measurement of purifying selection.^14^ With a score of 0.42, *SERPINB1* ranks among the top 30% of genes under stronger purifying selection in the human genome. Overall, our analysis indicates that SERPINB1 is under purifying selection, which suggests a critical, non-redundant role in the human immune system that we aim to unravel.

### SERPINB1 is ubiquitously expressed in immune cells, with the highest expression in monocytes

**Figure 2:**
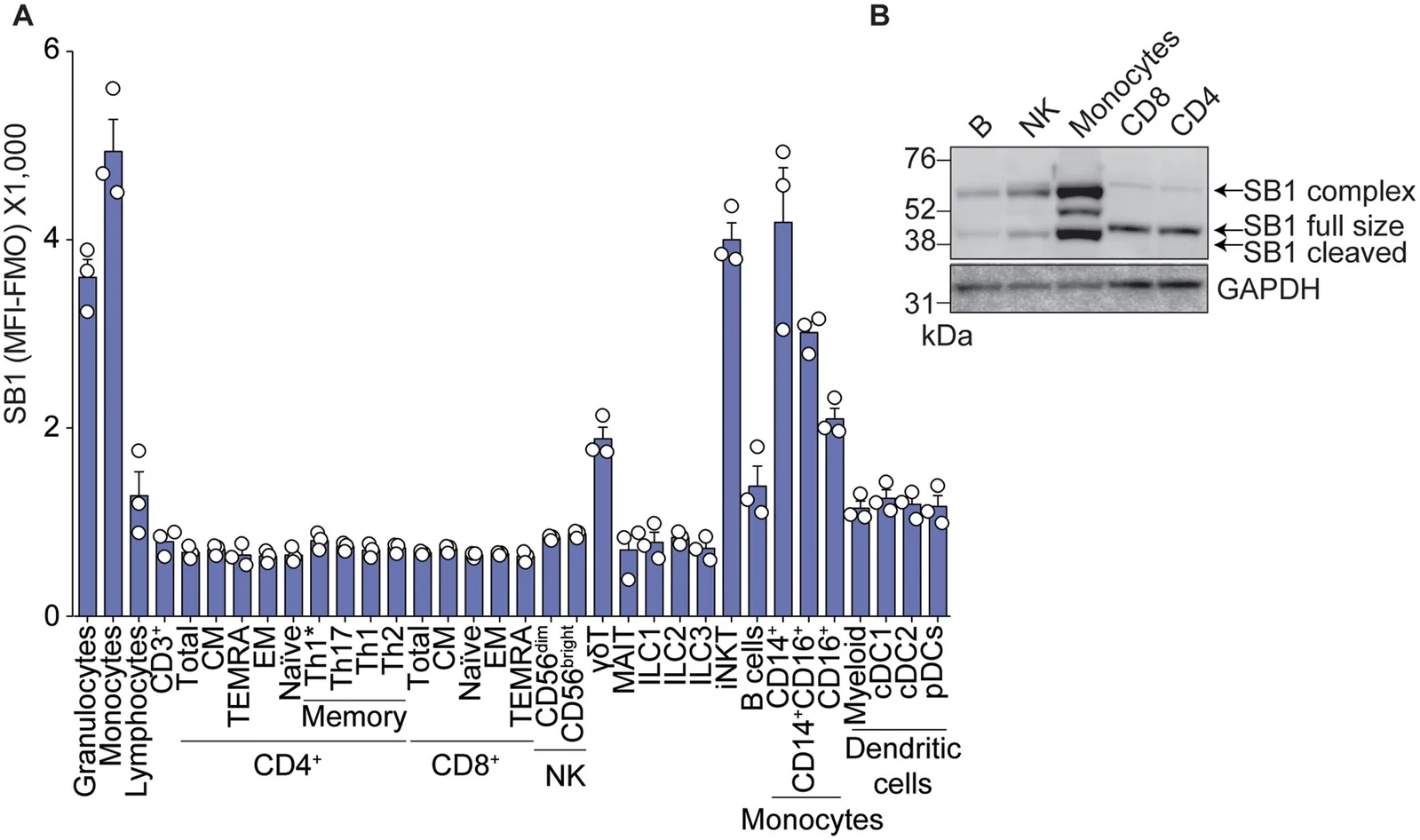
SERPINB1 expression in immune cells. (A) SERPINB1 levels in different immune populations as measured by FACS. The populations are indicated in the x-axis, and the Mean Fluorescence Intensity (MFI) minus the Fluorescence Minus One (FMO) for SERPINB1 signal is shown in the *y*-axis. The detailed gating strategy is shown in Fig. S1. (B) Western blot for expression of SERPINB1 in 5 main immune populations. GAPDH was used as a loading control.

After establishing that SERPINB1 is under purifying selection among critical immune genes, we sought to determine which immune cell populations express the highest levels of this protein to identify the human immune cell population in which SERPINB1 may play the most critical role. To do so, we assessed SERPINB1 expression in 34 immune cell populations by flow cytometry (Fig. 2A, S1, and S2). We observed that all immune cells analyzed showed SERPINB1 signal. To confirm that this signal corresponds to SERPINB1 expression and not background fluorescence, we sort-purified 5 main immune cell populations, namely B, NK, CD4^+^, CD8^+^ T cells, and monocytes, since they represent the spectrum of SERPINB1 expression by FACS (Fig. 2A). Consistent with our FACS results, we observed that all tested immune populations expressed SERPINB1 (Fig. 2B and S2). Furthermore, our Western blot analysis showed that SERPINB1’s function as a serine protease inhibitor was active at different levels in all these cells. SERPINB1 is a serine protease suicide inhibitor that forms a covalent bond with the proteases it inhibits. We observed a 2-band pattern, with the lower band corresponding to SERPINB1 and the upper band corresponding to SERPINB1 in complex with a serine protease. Another measure of SERPINB1 utilization as an intracellular protease inhibitor is the molecular weight of the SERPINB1 band. To inhibit serine proteases, SERPINB1 is proteolytically cleaved, reducing its molecular weight.^6^ Our Western blot in Fig. 2B shows that the molecular weight of SERPINB1 in B cells, NK cells, and monocytes is lower than in T cells (CD4^+^ and CD8^+^), indicating on the one hand that SERPINB1 is more active in the former and that the usage of SERPINB1 is complete, since no full-size SERPINB1 can be detected (Fig. 2B). This effect is especially evident in monocytes, which are the immune cell types with the highest expression of SERPINB1 (Fig. 2A, B). In addition to the bands corresponding to free SERPINB1 and its complexed form, the monocyte lane in Fig. 2B displays an additional intermediate band. Although this phenomenon has not been investigated in detail, this band may result from proteolytic cleavage of either SERPINB1 or its target proteases at a site exposed following covalent enzymatic inhibition. Alternatively, physical stress induced by conformational changes during complex formation could lead to partial fragmentation of one of the proteins. Overall, our data show that SERPINB1 is expressed in all human immune cells tested and that monocytes exhibit the highest expression and utilization of this molecule, suggesting that it may play a very important role in monocyte biology.

### SERPINB1 deficiency in THP-1 monocytic cells drives IL-1β production without affecting survival

**Figure 3:**
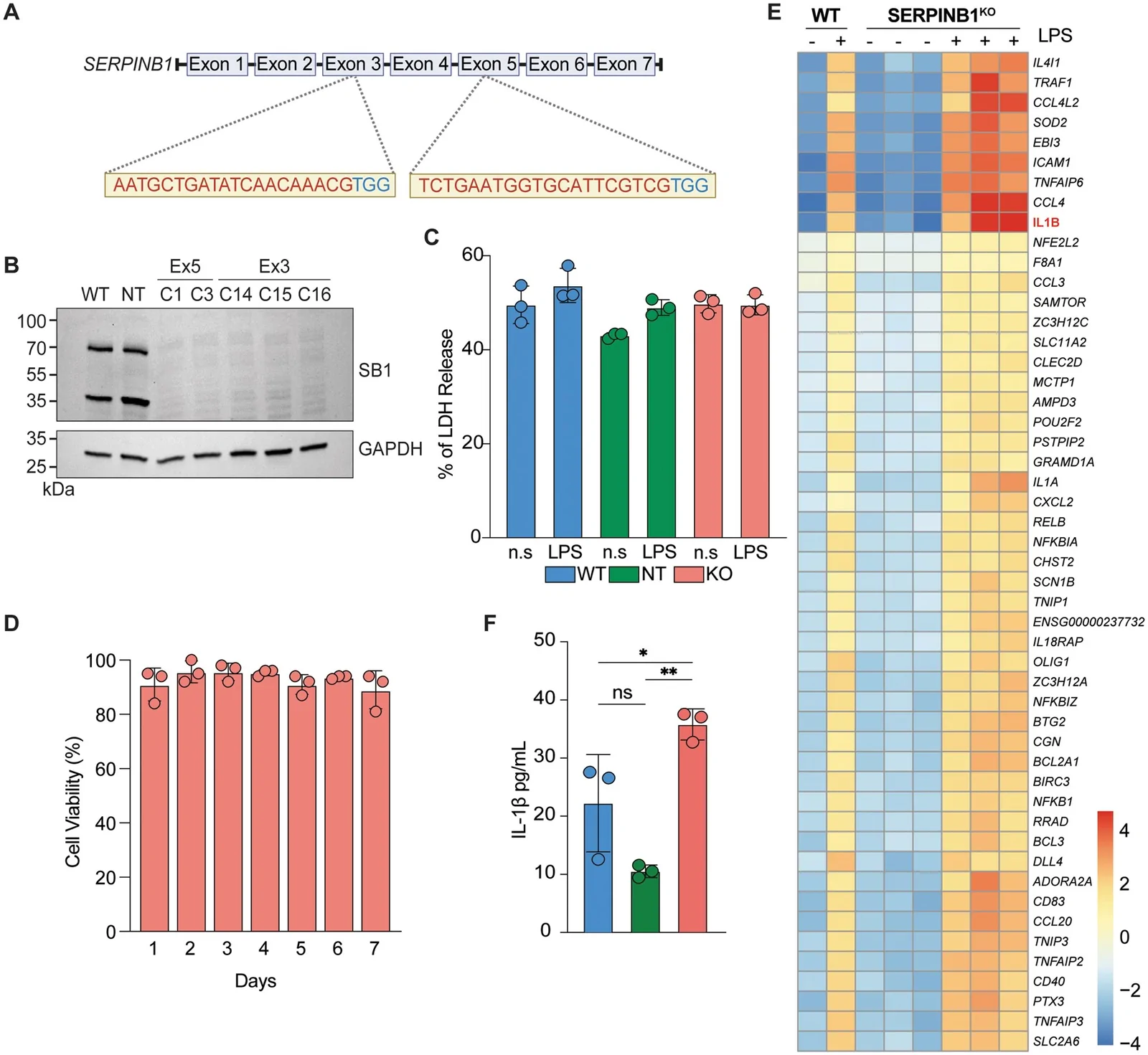
THP-1 SERPINB1 knockout generation, viability studies, and IL-1β production. (A) Schematic representation of *SERPINB1* in which each box represents an exon. Shown below are the sgRNA targets used to generate knockout clones, with the Protospacer Adjacent Motif (PAM) sequence indicated in blue. (B) Western blot showing SERPINB1 expression in the different THP-1 clones generated. GAPDH was used as a loading control. (C) LDH release assay of wild type (WT), non-target (NT), and knockout (KO) THP-1 cells, either treated or non-treated with LPS. (D) Cell viability measured by FACS of SERPINB1 knock-out cells during 7 d. (E) Heatmap of the top 50 upregulated genes in SERPINB1-deficient cells vs wild type after LPS stimulation. (F) IL-1β production by LPS-stimulated wild-type, non-target control, and SERPINB1-deficient THP-1 cells.

Since we showed that monocytes are the immune cells expressing the highest levels of SERPINB1 and observed that all present SERPINB1 is utilized in these cells, we set out to deepen our mechanistic understanding of these cells. To do so, we relied on THP-1 monocytic cells, which are useful tools for studying monocyte biology.^21^ We used CRISPR/Cas9 genome editing to generate THP-1 knockout cell lines using guides against exons 3 and 5 (out of the 7 exons that *SERPINB1* has) (Fig. 3A). In addition to knockout cells, we generated cell clones using a non-target single-guide RNA that does not target any area of the human genome, which we used as a control. After confirming that our clones were deficient in SERPINB1, we proceeded to identify alterations that the absence of SERPINB1 could cause in these cells (Fig. 3B). *Serpinb1a*, the predicted murine ortholog of human *SERPINB1*, plays a critical role in inhibiting intracellular proteases that, when unchecked, cause cell death.^10–12^^,^^22^ We first tested whether the absence of SERPINB1 altered THP-1 cell viability. We did this in 2 ways. We studied cell death by LDH release after LPS stimulation, which is designed to stress the cells and cause proteases from their granules to leak into the cytoplasm, as described before (Fig. 3C). We also measured viability in knockout cells over 7 d (Fig. 3D). Our results show that SERPINB1 deficiency does not alter THP-1 cell viability, suggesting that SERPINB1 has distinct functions from its murine ortholog. To identify additional pathways in which SERPINB1 may be involved, we performed bulk RNA-seq in wild-type cells and SERPINB1-deficient cells. We observed that the most upregulated genes in SERPINB1-deficient cells were proinflammatory, with *IL1B* among the most prominent (Fig. 3E and S2). To confirm these results, we stimulated wild-type, non-target control, and SERPINB1-knockout THP-1 cells with LPS and measured IL-1β production (Fig. 3F). We observed increased IL-1β production in SERPINB1-deficient cells, suggesting that SERPINB1 is an important regulator of monocytic proinflammatory responses. Overall, our findings suggest that, contrary to its murine counterpart, SERPINB1 does not play a role in cell viability but instead is a regulator of monocyte activation.

### Altered proteome in *SERPINB1*-deficient THP-1 cells

**Figure 4:**
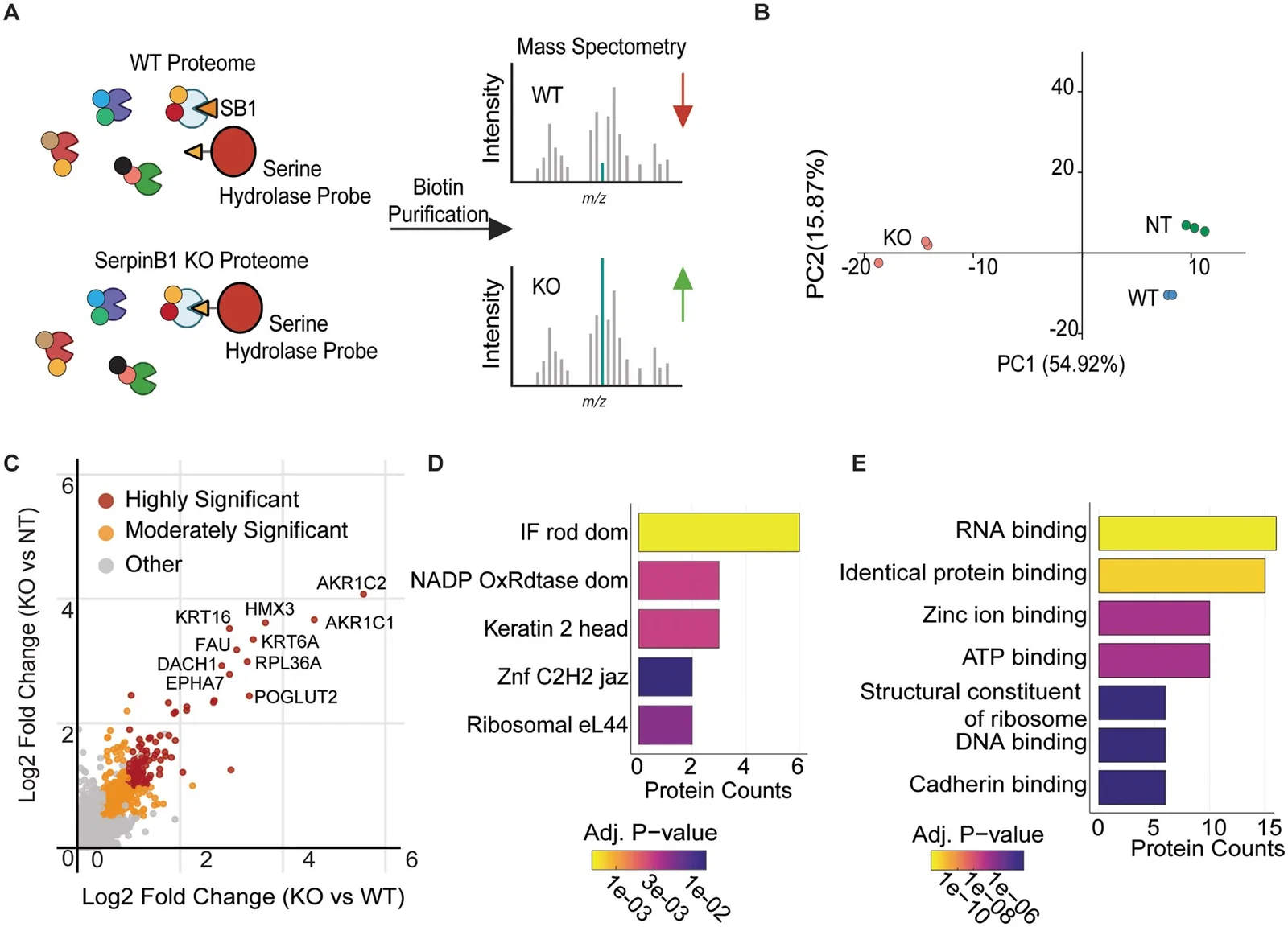
Activity-based proteomics profiling to identify SERPINB1-dependent pathways. (A) Schematic representation of the workflow for activity-based proteomic profiling (ABPP). (B) Dimensional reduction by principal component (PC) of the different cells used in this analysis. (C) Volcano plot highlighting the significantly increased molecules in the wild-type and non-target control cells when compared with the SERPINB1-deficient cells. The name of the top 10 identified proteins is indicated. (D) Domain and (E) pathway analysis using the proteins with the statistical threshold shown in panel C.

Our previous results suggest that human SERPINB1 has functions not previously described. To address this knowledge gap, we performed activity-based proteomic profiling (ABPP). ABPP is a technique designed to identify serine protease activity in a given sample. It uses a molecule containing a warhead that is targeted by serine proteases.^23^ After these enzymes react with the warheads, they remain covalently attached. The warheads are coupled to desthiobiotin, which allows affinity-based purification of these ABPP probes, along with the serine proteases they target and additional proteins that interact with these enzymes. By mass spectrometry, the identity of the immunoprecipitated proteins can be determined to identify pathways that are more active in the samples of interest (Fig. 4A).^23^ Given that SERPINB1 is a serine protease suicide inhibitor, we hypothesized that, in the absence of SERPINB1, certain serine protease-mediated pathways would be more active in knockout cells than in the wild type. We performed ABPP in cell lysates from wild-type, non-target controls, and SERPINB1-deficient THP-1 cells, followed by mass spectrometry. By principal component analysis (PCA), we observed that samples from wild-type and non-target controls cluster close together, whereas those from SERPINB1-deficient THP-1 cells have different properties than the previous 2 (Fig. 4B). These data indicate that SERPINB1 deficiency alters the overall enzymatic activity of THP-1 cells. Indeed, we identified numerous proteins with higher signal in SERPINB1-deficient cells than in wild-type or non-target control cells (Fig. 4C). Pathway and domain analyses suggest that multiple pathways are affected by SERPINB1, including ribosomal function and energy metabolism (Fig. 4D, E). Overall, our data show that SERPINB1 is involved in numerous pathways in monocytic cells.

## Discussion

The findings presented in this study highlight the critical and multifaceted role of SERPINB1 within the human immune system, particularly its prominent function in monocytes. Our evolutionary analysis revealed that *SERPINB1* is subject to purifying selection, with a CoNeS score comparable to that of genes known to cause autosomal recessive IEI when mutated.^13^ This evolutionary constraint highlights SERPINB1’s indispensable and non-redundant role in immune homeostasis, underscoring the potentially deleterious consequences of its dysfunction. The broad expression of SERPINB1 across diverse immune cell subsets, as demonstrated by flow cytometry, coupled with Western blot analyses showing active protease inhibition predominantly in monocytes, consistent with previous reports,^4^^,^^24–26^ suggests a heightened demand for intracellular regulation of serine proteases in these cells. This pattern likely reflects monocytes’ complex roles in immune surveillance, inflammation, and pathogen clearance, in which tight control of proteolytic activity is essential to prevent unwanted cell damage or dysregulated inflammatory responses.^27^^,^^28^ Our expression analysis reveals significant differences between human SERPINB1 and its murine counterpart. Although Serpinb1a expression has been extensively characterized in mouse monocytes, macrophages, and neutrophils, we identified notable species-specific distinctions: in mice, Serpinb1a is absent in resting T cells and is induced only *in vitro* during T_H_17 differentiation of CD4^+^ cells, whereas in humans, both CD4^+^ and CD8^+^ T cells exhibit baseline SERPINB1 expression (Fig. 2).^16^^,^^29^ Moreover, unlike murine Serpinb1a, which is restricted to neutrophils, monocytes, *in vitro* differentiated T_H_17 CD4^+^ T cells, γδ T cells, and iNKT cells, we provide the first evidence that SERPINB1 is broadly expressed at the protein level across diverse human immune cell populations, thereby expanding the current understanding of its immune distribution.^29–33^

Interestingly, our functional data contrast with previous murine models and recent findings reported by Choi and colleagues, where shRNA-mediated SERPINB1 knockdown in THP-1 cells increased caspase-dependent IL-1β secretion without altering protein synthesis and reduced cell viability.^7^^,^^10–12^^,^^22^ In contrast, we used CRISPR-Cas9 gene editing, a more precise and effective approach to knock out SERPINB1, and observed no differences in THP-1 cell viability and an increase in *IL1B* transcription (Fig. 3). This discrepancy suggests that the divergent outcomes may be due to methodological differences or underlying biological variation related to the different levels of SERPINB1 in these engineered cells. Beyond confirming enhanced IL-1β secretion after LPS stimulation, consistent with this previous report,^7^ our transcriptomic profiling revealed a broader proinflammatory signature characterized by upregulation of genes such as *TRAF1*, *CCL4*, *EBI3*, and *ICAM1* (Fig. 3E). These findings indicate that SERPINB1 deficiency affects inflammatory pathways beyond caspase and inflammasome activation, complementing previous work and broadening the scope of its regulatory influence.^7^ Further evidence for the existence of yet undiscovered roles of SERPINB1 comes from our ABPP study. To the best of our knowledge, this study represents the first application of ABPP to investigate the enzymatic landscape in the context of SERPINB1 deficiency. ABPP uncovered alterations in pathways related to ribosomal function and cellular metabolism among others (Fig. 4). Supporting these findings, transcriptomic data showed upregulation of metabolic genes, including *IL4I1* and *SOD2*, highlighting a previously unrecognized link between SERPINB1 and metabolic regulation (Fig. 3E). Furthermore, ABPP identified enriched ribosomal proteins and proteins harboring structural constituents of ribosome domains in the SERPINB1-deficient cells, suggesting for the first time a novel role for SERPINB1 in ribosome biology. Collectively, these pleiotropic effects imply that SERPINB1 orchestrates a previously unrecognized complex network that intersects protease inhibition, inflammatory signaling, metabolic adaptation, and translational control to maintain monocyte immune homeostasis and prevent excessive inflammation, in contrast to the narrower caspase-dependent IL-1β secretion function previously described.^7^ Our findings warrant validation using primary human monocytes to confirm the results obtained with THP-1 monocytic cell lines, which may not fully replicate SERPINB1 function in monocytes and macrophages. However, collectively, our results advance the conceptual framework of SERPINB1 as a multifaceted intracellular regulator. Future mechanistic studies dissecting its interactions with specific proteases, metabolic enzymes, and ribosomal components will be critical to fully elucidate its role and therapeutic potential in inflammatory and immune-mediated diseases.

## Supplementary Material

vlag053_Supplementary_Data

## Data Availability

The data generated during this study are available in the manuscript, in the supplementary material, and in the MassIVE repository (accession number MSV000102433). Any additional data can be requested from the corresponding author upon reasonable request.

## References

[1] Zeng W , Silverman GA, Remold-O’Donnell E. Structure and sequence of human M/NEI (monocyte/neutrophil elastase inhibitor), an Ov-serpin family gene. Gene. 1998;213:179–187. 10.1016/s0378-1119(98)00189-9 9630619

[2] Silverman GA et al Human clade B serpins (ov-serpins) belong to a cohort of evolutionarily dispersed intracellular proteinase inhibitor clades that protect cells from promiscuous proteolysis. Cell Mol Life Sci. 2004;61:301–325. 10.1007/s00018-003-3240-3 PMC11138797 14770295

[3] Kaiserman D , Bird PI. Analysis of vertebrate genomes suggests a new model for clade B serpin evolution. BMC Genomics. 2005;6:167. 10.1186/1471-2164-6-167 PMC1308813 16305753

[4] Remold-O’Donnell E , Chin J, Alberts M. Sequence and molecular characterization of human monocyte/neutrophil elastase inhibitor. Proc Natl Acad Sci U S A. 1992;89:5635–5639. 10.1073/pnas.89.12.5635 PMC49347 1376927

[5] Torriglia A , Martin E, Jaadane I. The hidden side of SERPINB1/Leukocyte elastase inhibitor. Semin Cell Dev Biol. 2017;62:178–186. 10.1016/j.semcdb.2016.07.010 PMC5610702 27422329

[6] Cooley J , Takayama TK, Shapiro SD, Schechter NM, Remold-O’Donnell E. The serpin MNEI inhibits elastase-like and chymotrypsin-like serine proteases through efficient reactions at two active sites. Biochemistry. 2001;40:15762–15770. 10.1021/bi0113925 11747453

[7] Choi YJ et al SERPINB1-mediated checkpoint of inflammatory caspase activation. Nat Immunol. 2019;20:276–287. 10.1038/s41590-018-0303-z PMC6450391 30692621

[8] Benarafa C , Cooley J, Zeng W, Bird PI, Remold-O’Donnell E. Characterization of four murine homologs of the human ov-serpin monocyte neutrophil elastase inhibitor MNEI (SERPINB1). J Biol Chem. 2002;277:42028–42033. 10.1074/jbc.M207080200 12189154

[9] Benarafa C. The SerpinB1 knockout mouse a model for studying neutrophil protease regulation in homeostasis and inflammation. Methods Enzymol. 2011;499:135–148. 10.1016/B978-0-12-386471-0.00007-9 21683252

[10] Benarafa C et al SerpinB1 protects the mature neutrophil reserve in the bone marrow. J Leukoc Biol. 2011;90:21–29. 10.1189/jlb.0810461 PMC3114599 21248149

[11] Baumann M , Pham CT, Benarafa C. SerpinB1 is critical for neutrophil survival through cell-autonomous inhibition of cathepsin G. Blood. 2013;121:3900–3907. 10.1182/blood-2012-09-455022 PMC3650706 23532733

[12] Loison F et al Proteinase 3-dependent caspase-3 cleavage modulates neutrophil death and inflammation. J Clin Invest. 2014;124:4445–4458. 10.1172/JCI76246 PMC4191030 25180606

[13] Rapaport F et al Negative selection on human genes underlying inborn errors depends on disease outcome and both the mode and mechanism of inheritance. Proc Natl Acad Sci U S A. 2021;118:e2001248118. 10.1073/pnas.2001248118 PMC7826345 33408250

[14] Eilertson KE , Booth JG, Bustamante CD. SnIPRE: selection inference using a Poisson random effects model. PLoS Comput Biol. 2012;8:e1002806. 10.1371/journal.pcbi.1002806 PMC3516574 23236270

[15] Sedelies KA et al Discordant regulation of granzyme H and granzyme B expression in human lymphocytes. J Biol Chem. 2004;279:26581–26587. 10.1074/jbc.M312481200 15069086

[16] Hou L et al SerpinB1 controls encephalitogenic T helper cells in neuroinflammation. Proc Natl Acad Sci U S A. 2019;116:20635–20643. 10.1073/pnas.1905762116 PMC6789640 31548399

[17] Notarangelo LD , Bacchetta R, Casanova JL, Su HC. Human inborn errors of immunity: an expanding universe. Sci Immunol. 2020;5:eabb1662. 10.1126/sciimmunol.abb1662 PMC7647049 32651211

[18] Poli MC et al Human inborn errors of immunity: 2024 update on the classification from the International Union of Immunological Societies Expert Committee. J Hum Immun. 2025;1:e20250003. 10.70962/jhi.20250003 PMC12829761 41608114

[19] Deschamps M et al Genomic signatures of selective pressures and introgression from Archaic Hominins at Human Innate Immunity Genes. Am J Hum Genet. 2016;98:5–21. 10.1016/j.ajhg.2015.11.014 PMC4716683 26748513

[20] Quintana-Murci L , Clark AG. Population genetic tools for dissecting innate immunity in humans. Nat Rev Immunol. 2013;13:280–293. 10.1038/nri3421 PMC4015519 23470320

[21] Chanput W , Mes JJ, Wichers HJ. THP-1 cell line: an in vitro cell model for immune modulation approach. Int Immunopharmacol. 2014;23:37–45. 10.1016/j.intimp.2014.08.002 25130606

[22] Benarafa C , Priebe GP, Remold-O’Donnell E. The neutrophil serine protease inhibitor serpinb1 preserves lung defense functions in *Pseudomonas aeruginosa* infection. J Exp Med. 2007;204:1901–1909. 10.1084/jem.20070494 PMC2118684 17664292

[23] Porta EOJ , Steel PG. Activity-based protein profiling: a graphical review. Curr Res Pharmacol Drug Discov. 2023;5:100164. 10.1016/j.crphar.2023.100164 PMC10484978 37692766

[24] Remold-O’Donnell E. A fast-acting elastase inhibitor in human monocytes. J Exp Med. 1985;162:2142–2155. 10.1084/jem.162.6.2142 PMC2187972 3906019

[25] Remold-O’Donnell E , Nixon JC, Rose RM. Elastase inhibitor. Characterization of the human elastase inhibitor molecule associated with monocytes, macrophages, and neutrophils. J. Exp. Med. 1989;169:1071–1086. 10.1084/jem.169.3.1071 PMC2189272 2926322

[26] Sugimori T , Cooley J, Hoidal JR, Remold-O’Donnell E. Inhibitory properties of recombinant human monocyte/neutrophil elastase inhibitor. Am J Respir Cell Mol Biol. 1995;13:314–322. 10.1165/ajrcmb.13.3.7654387 7654387

[27] Murray PJ. Immune regulation by monocytes. Semin. Immunol. 2018;35:12–18. 10.1016/j.smim.2017.12.005 29290545

[28] Parihar A , Eubank TD, Doseff AI. Monocytes and macrophages regulate immunity through dynamic networks of survival and cell death. J Innate Immun. 2010;2:204–215. 10.1159/000296507 PMC2956013 20375558

[29] Zhao P , Hou L, Farley K, Sundrud MS, Remold-O’Donnell E. SerpinB1 regulates homeostatic expansion of IL-17+ gamma-delta and CD4+ Th17 cells. J Leukoc Biol. 2014;95:521–530. 10.1189/jlb.0613331 PMC3923080 24249741

[30] Narayan K , et al; Immunological Genome Project Consortium. Intrathymic programming of effector fates in three molecularly distinct γδ T cell subtypes. Nat Immunol. 2012;13:511–518. 10.1038/ni.2247 PMC3427768 22473038

[31] Engel I et al Innate-like functions of natural killer T cell subsets result from highly divergent gene programs. Nat Immunol. 2016;17:728–739. 10.1038/ni.3437 PMC4944658 27089380

[32] Georgiev H , Ravens I, Benarafa C, Förster R, Bernhardt G. Distinct gene expression patterns correlate with developmental and functional traits of iNKT subsets. Nat Commun. 2016;7:13116. 10.1038/ncomms13116 PMC5062562 27721447

[33] Leborgne NGF , Taddeo A, Freigang S, Benarafa C. Serpinb1a is dispensable for the development and cytokine response of invariant natural killer T cell subsets. Front Immunol. 2020;11:562587. 10.3389/fimmu.2020.562587 PMC7686238 33262755

