# Supplementary material for: SERPINB1 as a critical regulator of inflammation and protease activity in human monocytic cells
Source: Immunohorizons. 2026 Sep 24;10(9):vlag053. doi: 10.1093/immhor/vlag053 (PMC13612166; doi:10.1093/immhor/vlag053)
Supplement: Supplementary file 1 [file vlag053_supplementary_data.zip › THP1-SB1-SOM.pdf]

SUPPLEMENTARY MATERIAL

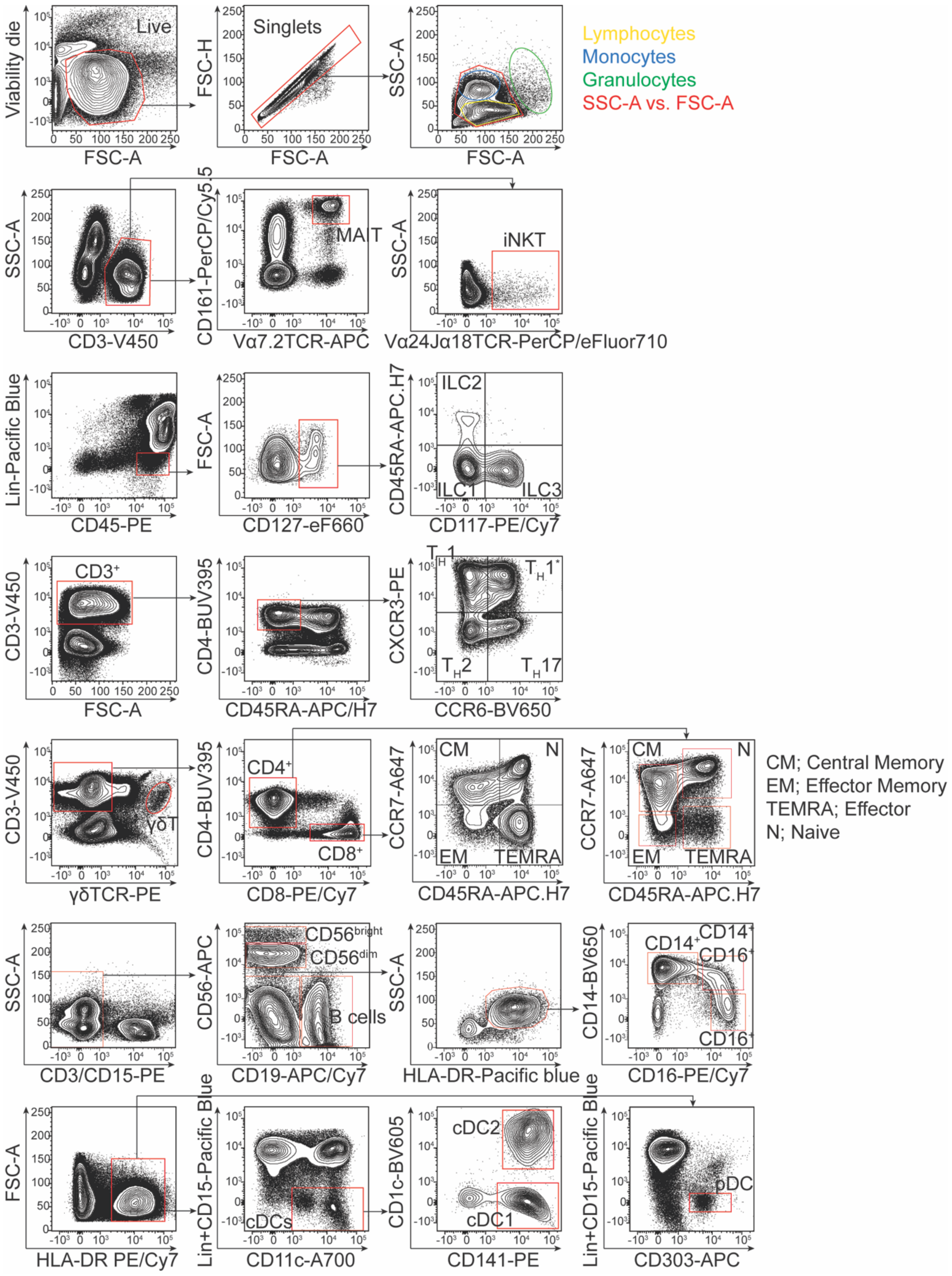

Supplementary Figure 1: Gating strategy for SERPINB1 expression in immune cells.

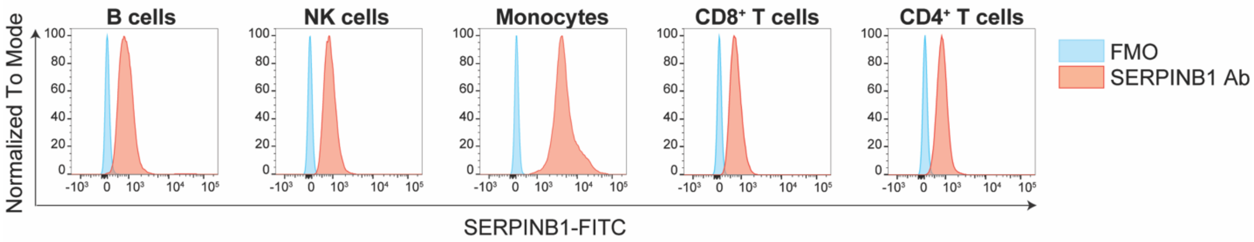

Supplementary Figure 2: SERPINB1 and Fluorescence Minus One control. Representative plots showing SERPINB1 levels detected with an antibody conjugated to FITC and the signal for the FMO control in the same population. The names of the populations are indicated above the histograms and correspond to those analyzed by Western blot in Figure 2C.

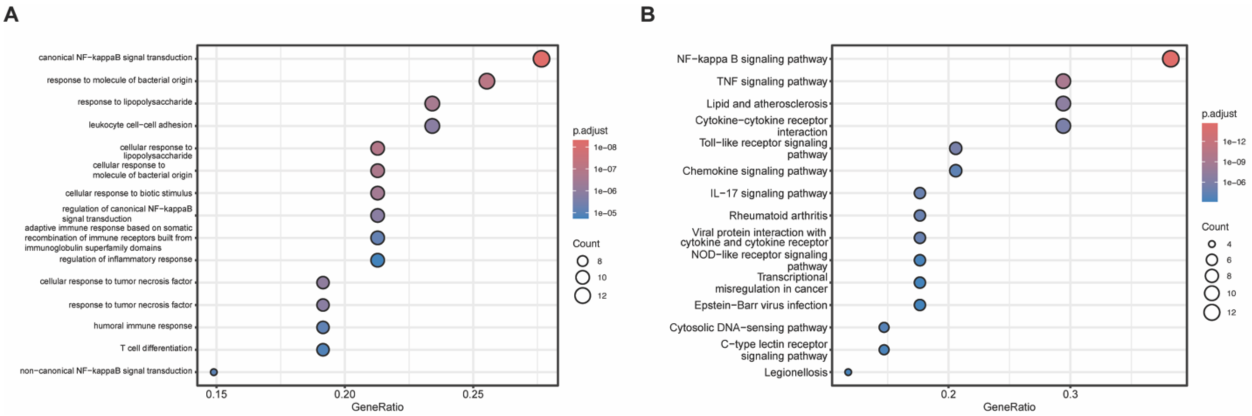

Supplementary Figure 3: Pathway analysis from RNA seq.

Pathway analysis of the top 50 upregulated genes by RNAseq from wild type and SERPINB1-deficient THP-1 cells treated or untreated with LPS using **A)** GO or **B)** KEGG
